# Multiple fragmented habitat-patch use in an urban breeding passerine, the Short-toed Treecreeper

**DOI:** 10.1371/journal.pone.0227731

**Published:** 2020-01-14

**Authors:** Katherine R. S. Snell, Rie B. E. Jensen, Troels E. Ortvad, Mikkel Willemoes, Kasper Thorup

**Affiliations:** Center for Macroecology, Evolution and Climate, Natural History Museum of Denmark, University of Copenhagen, Copenhagen, Denmark; Universitat Autònoma de Barcelona, SPAIN

## Abstract

Individual responses of wild birds to fragmented habitat have rarely been studied, despite large-scale habitat fragmentation and biodiversity loss resulting from widespread urbanisation. We investigated the spatial ecology of the Short-toed Treecreeper *Certhia brachydactyla*, a tiny, resident, woodland passerine that has recently colonised city parks at the northern extent of its range. High resolution spatiotemporal movements of this obligate tree-living species were determined using radio telemetry within the urbanized matrix of city parks in Copenhagen, Denmark. We identified regular edge crossing behaviour, novel in woodland birds. While low numbers of individuals precluded a comprehensive characterisation of home range for this population, we were able to describe a consistent behaviour which has consequences for our understanding of animal movement in urban ecosystems. We report that treecreepers move freely, and apparently do so regularly, between isolated habitat patches. This behaviour is a possible driver of the range expansion in this species and may contribute to rapid dispersal capabilities in certain avian species, including Short-toed Treecreepers, into northern Europe. Alternatively, these behaviours might be common and/or provide an adaptive advantage for birds utilising matrix habitats, for example within urban ecosystems.

## Introduction

Increasing urbanisation globally, with associated habitat fragmentation and loss, has prompted a wealth of studies of effects of urban sprawl in birds [1, 2]. Urbanisation is one of the fastest growing land use changes, with consequential impacts on biodiversity (extinction, speciation and distribution) across taxa [3, 4]. Urban avian assemblages are affected by a variety of factors, such as vegetation structure and diversity, human disturbance and anthropogenic provision of resources [1, 2, 5–7].

Fragmentation of habitat limits dispersal and colonisation even in species as mobile as birds [8]. The influence of matrix habitat as a result of fragmentation from the perspective of population dynamics and species assemblage has been extensively studied ([8–13] and papers therein). Behaviour at the individual level is rarely considered [4, 9, 14] but for example, Moore et al. [15] found very poor obligate crossing ability in tropical rainforest specialists, and Black-capped Chickadees *Parus atricapillus* were less likely to cross open areas between woodlands [16, 17]. In an urban setting, Evans et al. [1] concluded that fragmentation of habitats frequently influences avian assemblages. Furthermore, a feeder visitation study reported a negative effect of increased urbanisation on functional movements [18]. While much work is devoted to the study of urbanisation, and its effects on demographic parameters and species assemblages (reviewed in [1]), empirical data of how vagile species interact with this landscape are generally lacking.

Short-toed Treecreepers are obligate tree-living passerines resident predominantly in central Europe in low densities [19, 20]. Unlike the Eurasian Treecreeper *Certhia familiaris*, the Short-toed Treecreeper has exhibited rapid range expansion and has only recently become established in Denmark (first documented in 1930) at the northernmost extent of its range [21, 22]. This northward range expansion is likely caused by a combination of dispersal/ movement events and climate drivers [23]. *Certhia* species are not strong fliers although the Eurasian Treecreeper is migratory [24]. The two species prefer different habitats; whereas the Eurasian Treecreeper is apparently dependant on young, dense woodland, the Short-toed Treecreeper utilises both urban and mature forest landscapes [25, 26]. Short-toed Treecreepers feed, nest, and roost exclusively on the trunks or branches of trees [27]. They specialise in mature rough barked species in edge habitats [27].

Urban wooded habitats are generally small scale: a fragmented matrix of parks and tree-lined corridors [28, 29], typically 1–100 ha separated by tens to hundreds of metres of hard structures (roads and buildings). These urban woodlands are often considered islands within the urban landscape [10, 30–32]. Urban parks are characterised by highly modified spaces, low tree density, large numbers of exotics and a mixture of non-native and native genera [28, 30]. Mature trees are heavily managed, particularly in the canopy [28]. In Northern Europe, which is characterised by the largely temperate climate and frost-resistant broadleaved deciduous native forests, mature trees are predominantly hardwood, with a large proportion reported in Danish cities [33, 34]. In addition to parks and cemeteries, city spaces include significant numbers of street trees [34, 35]. For example, in Aarhus, Denmark, one third of all trees are street trees [34].

We explicitly investigate spatial behaviours at the individual level [32]. Even in the era of rapid development of tracking technologies, for small birds tag-size limits high spatial- and temporal-resolution tracking to radio telemetry [36, 37] which is particularly valuable in cryptic species. Tracking provides a systematic unbiased tool to document space utilisation of individuals [36, 38] to identify habitat attributes. Previous studies attempting to track urban breeding birds are generally restricted to large species (e.g. raptors, pigeons and parakeets: [39–43]), and studies of songbirds within an urbanised environment have focused on post-natal dispersal [44–47]. Here, we used radio telemetry to identify the structural habitat of a typically woodland bird, now breeding in the highly urbanised area of central Copenhagen, and characterise movements of Short-toed Treecreepers in the built landscape.

## Materials and methods

In the pre-breeding periods (2016 & 2017), four Short-toed Treecreepers were caught in Fælledparken in the centre of Copenhagen, using play-back and continuously monitored mist-nets. Birds were full-grown males (later determined from song). They were ringed with a unique metal and colour ring and fitted with a tail-mounted radio tag (0.35g, 16ms pulse, 48bpm pulse rate; Biotrack, UK; all procedures followed national ethical guidelines). The tag and rings accounted for <5% of the birds’ body mass (8.25–9.0g), following [48]. Tag attachment was designed to be temporary, i.e. until the single tail feather was moulted and replaced; as such birds were tracked until the tag was dropped (3–8days). Positions were obtained using a Sika Receiver and Flexible Yagi handheld antenna throughout the daylight period allowing at least one hour separation between positions, to account for the inherent spatial auto-correlation of movement data (see positional data) [36, 38]. If the bird was obscured by vegetation, the tree positon was determined by triangulation. For 62% of positions, visual confirmation of the bird was possible, once located by telemetry.

To illustrate utilization areas, 100% minimum convex polygons (MCPs) were calculated (adehabitat R package; R v3.5.3 [49, 50]). Supporting visual-only re-sighting data from one individual ringed in 2016 was included in the 2017 dataset (n = 5 opportunistic positions within the same pre-breeding period) and years were treated independently for analysis.

**Fig 1:**
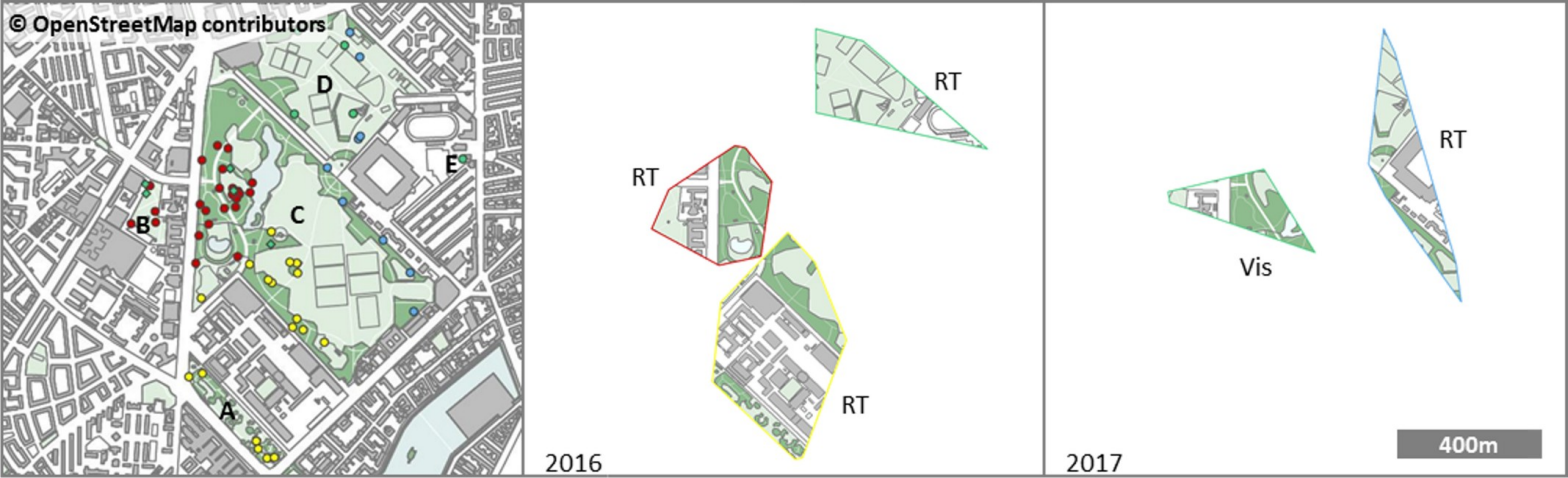
Positions of tracked birds in central Copenhagen, Denmark. Positional fixes derived from ● Radio Telemetry (RT) and ◆ Visual-only observations (Vis) for the four individual Short-toed Treecreepers indicated by colour of colour ring. Minimum convex polygon (100%) is given to illustrate known area use in 2016 and 2017. ‘Built-up’ areas: grey; ‘urban park’ includes grass: pale green, trees: dark green, lakes: blue; and all ‘roads’ and other hard surfaces: white. Isolated habitat patches are labelled: **A** Amorparken, **B** Universitetsparken, **C** Fælledparken S, **D** Fælledparken N and **E** Sankt Jakobs Kirkehave. All panels at the same scale and extent.

High resolution land use data were derived from Open StreetMap (OSM) categorised polygons [51, 52]. We aggregated land use into three categories: ‘urban park’, ‘built-up’ and ‘roads’ from OSM classification tags (S1 Table), and extracted land use by area within each MCP. We *a priori* defined ‘urban park’ areas with mature stands of trees as suitable habitat for the Short-toed Treecreepers [27]. Discrete habitat patches were defined as contiguous areas of ‘urban park’ separated by ‘built-up’ areas and ‘roads’: here Amorparken, Universitetsparken, Fælledparken S, Fælledparken N and Sankt Jakobs Kirkehave (Fig 1). To demonstrate movements within and between separated habitat blocks, straight line distances between positions relative to the first telemetry-derived position were calculated using deg.dist() function (fossil R package [53]).

Tree cover data were included in the maps for figurative reference. Mature trees within the study area were identified and mapped within the study area, and minimum flight distance between discrete urban park fragments in each bird’s utilization area was measured as the shortest straight-line distance between closest mature trees. Tree preference was obtained in 2016 for the two individuals with more than 10 visually confirmed positions as the mean measures of girth (CBH), bark furrow-depth (mean of five random positions around CBH measure) and a proxy for local density (distance to nearest mature tree).

### Ethics statement

This study was carried out in strict accordance with necessary national and local permissions and guidelines. Capture with mist-nets and play-back, ringing, single-tail feather mounted radio-tagging and tracking was approved by the steering committee of the Danish Nature Agency by permission to the Copenhagen Bird Ringing Centre (J.nr. SN 302–009). Landowners’ permissions were obtained for fieldwork at these sites.

## Results

**Fig 2:**
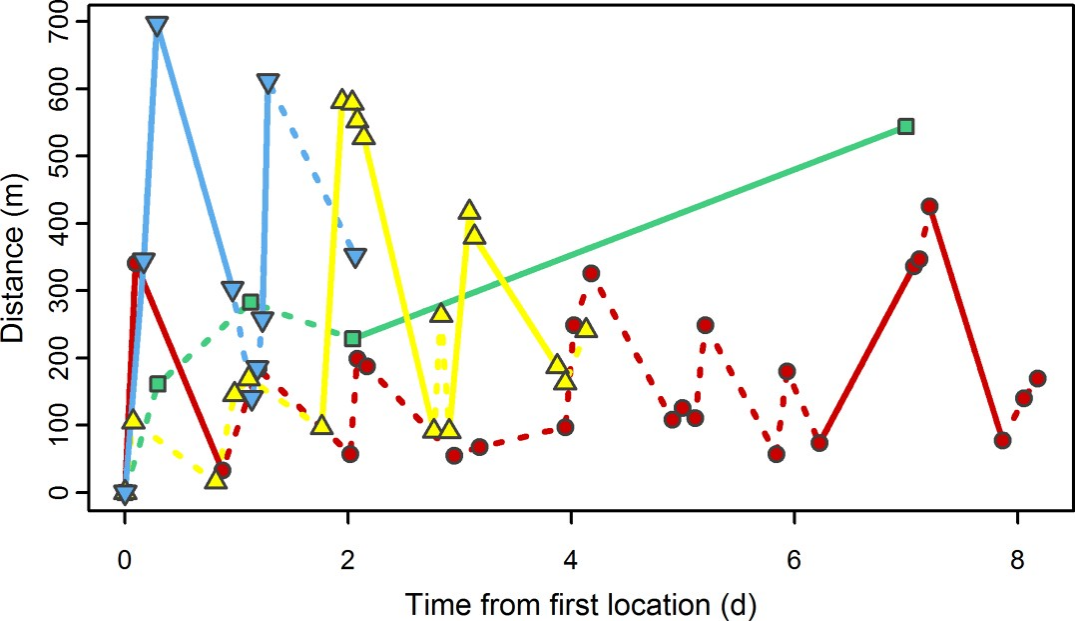
Distance between radio-telemetry positions over the tracking period. Movement between consecutive positions within the same habitat fragment represented by dashed lines (**----**) and movements between positions in different habitat fragments represented by a solid line (**——**). Distance (m) is the straight line distance of each position from the first independent position derived by radio telemetry. Time in days (d) is the time passed since the first position. Capture and tagging positions were not used as the reference location as it may have been confounded by the use of playback. Colours and symbols indicate individuals by colour ring: red ●, green ■, yellow ▲, blue ▼.

All birds were observed in at least two areas of suitable habitat separated by built-up land of 32 to 200m minimum distance (Fig 1 and S2 Table). Positions indicate multiple movements between discrete habitat blocks over the duration of the study period (Fig 2). Where high temporal resolution positions were obtained, these movements appear to be regular diurnal translocation behaviours (Fig 2). Positions were located in park areas separated by main roads, carparks or university/hospital buildings (ca. 5 stories), typical of a European city non-residential area. Where habitat patches are separated by multi-storey buildings birds may have flown directly across (observed in 2017, pers. comm. M. Thorup), or undertaken a route that circumnavigated buildings potentially crossing shorter distances between habitat patches (i.e. utilising minimum distance between patches). Calculated MCPs (S2 Table) are indicative of the land-use matrix in the known utilisation area, and although not a typically defined home range, areas ranged between 9.8 and 20.6ha, of which 5.8 to 7.7ha was urban park land. Individuals, when located, were in large, lone-standing trees with deep furrows (girth 2.4±0.5m, distance to nearest tree 6.0±0.6m and furrow depth 67±2.7mm (n = 28, ±SD)).

## Discussion

Our study demonstrates regular movement of individual Short-toed Treecreepers among isolated areas of habitat across a fragmented, urban landscape. Our low population density, and hence low sample size precludes a comprehensive assessment of home range and we are conservative in identifying any patterns of behavioural ecology. However, characterisation of spatial dynamics in urban fragmented habitat at the individual level is rare and has not previously been established at this scale for free-flying passerines.

The flexible and adaptive behavioural pattern observed here may explain not only the rapid and recent expansion of their range, but also the utilisation of urban habitats [32]. The limited but systematic non-biased data in our study demonstrate that birds not only made multiple flights from one fragment to another, crossing high-traffic roads or built-up areas, but did so apparently routinely as part of a diurnal pattern. The use of lone-standing, deeply-furrowed trees in the urban environment is typical of habitat for this species in its core range (e.g. Spain [27]). Tree-lined streets or ‘ecological corridors’ in Denmark, with their propensity for young trees, are invariably unsuitable breeding or foraging habitat for our study species [27]; however they may facilitate movement (regular or dispersive) within the urban matrix [35, 47, 54]. The intra-patch flights of up to 200m were neither confined to contiguous habitat nor, in all cases, reliant upon these corridors. This is perhaps surprising given that previous studies found that birds were (i) reluctant to leave sites when separated by urban development [55], (ii) incurred apparent survival/productivity costs when switching patches [56], (iii) gap-crossing tendency is apparently allometric [57] and (iv) that insectivores, in particular, were unlikely to penetrate edges [58]. Our findings do not corroborate the idea that energetic cost and vulnerability to predation (from aerial predators) means that small songbirds are unlikely to undertake edge crossing behaviour [16, 17, 59].

Our study highlights the potential implication that an overestimate of population size may be inadvertently derived from visual-only observations with the inaccurate assumption that they are sedentary or averse to crossing built up landscapes. Conservation and management of habit for urban wildlife is an important emerging field, and demographic parameters of species are essential [10, 60]. In this species, and potentially other songbirds, that move between patches of habitat, it is conceivable that the same individual is recorded as multiple apparent territories.

Technology is fundamental to the understanding of spatial characteristics of key utilisation areas, generally unbiased by type II error, and therefore the limitations of investigating songbirds in urban environments must be implicit. For animals with a body mass of less than 50g, fully independent spatial data is still confined to radio telemetry, although there is much interest in advancing functionality with this technology [37, 61]. Apparent sources of interference identified from this study alone include: aviation; reflections from buildings (as described in [36]) and transmitting radio-frequency interference which can lead to abrupt signal masking or indeterminate positions. We identified attenuation from galvanised chain-link fences, tree trunks and dense vegetation (latter described in [62]), and competing pulsed transmissions on the same frequency. In combination, methodology must account for these limitations of tracking in urban environments to be able to inform spatial ecology of home ranges and resource partitioning.

There is a wealth of literature addressing habitat fragmentation from the perspective of population dynamics and species assemblage ([9, 29] and papers therein). These studies range from pattern-based conceptual models to species-orientated responses to their environment. Field studies invariably rely on methodologically limited movement data for the questions addressed in our study, i.e. point counts, citizen science monitoring programs, proximity loggers or colour ring resightings [18, 55, 56, 63]. However, while they provide complementary insights into processes of species abundance and assemblage, below the population level, the individual is rarely considered [4, 9, 12, 14]. While our sample size was necessarily low, the same pattern of edge-crossing was recorded in all individuals. Even conservatively, we can conclude that this capability exists in this population. The capacity to utilise fragmented habitat by Short-toed Treecreepers may provide a complementary driver of rapid range expansion, and such behaviour may enable species to colonise urban environments or persist in rapidly changing landscapes [3, 64, 65]. Our study presents novel insights into how some birds interact with the heterogeneity of the built environment and urban greenspaces, with potential implications for understanding the mechanism of range expansion.

## Supporting information

S1 Table Land use data definitions. Land use data derived from Open StreetMap (OSM) categorised polygons, classification tags and definitions.
(PDF)

S2 Table Spatial data acquired for study birds. Total number of positions acquired for each of four individual Short-toed Treecreepers in Copenhagen, Denmark, the calculated 100% minimum convex polygon (MCP) of known area use, and total area of ‘urban park’ and ‘road’ (calculated from Open StreetMap land use) within. Minimum Distance (Min. dist) between mature trees in park fragments used by each individual (as calculated from satellite images).
(PDF)
