# Supplementary material for: Multiple fragmented habitat-patch use in an urban breeding passerine, the Short-toed Treecreeper
Source: PLoS One. 2020 Jan 14;15(1):e0227731. doi: 10.1371/journal.pone.0227731 (PMC6959574; doi:10.1371/journal.pone.0227731)
Supplement: S1 Table — Land use data derived from Open StreetMap (OSM) categorised polygons, classification tags and definitions. (PDF) [file pone.0227731.s001.pdf]

S1 Table. Land use data definitions

Land Use	OSM classification	OSM Definition
urban park	leisure=park	open areas for recreational use in a semi-natural state with grass, trees and bushes (note: here all parks contained mature trees)
	landuse=forest	managed or plantation woodland
built-up	buildings=*	university, residential, sporting facilities
	landuse=construction	construction sites
roads	highway=*	all paved highways and adjoining pedestrian/cycle facilities, driveways (note: not footpaths intersecting parks)
	amenity=parking	carparks

Land use data derived from Open StreetMap (OSM) categorised polygons, classification tags and definitions.
