# Supplementary material for: Multiple fragmented habitat-patch use in an urban breeding passerine, the Short-toed Treecreeper
Source: PLoS One. 2020 Jan 14;15(1):e0227731. doi: 10.1371/journal.pone.0227731 (PMC6959574; doi:10.1371/journal.pone.0227731)
Supplement: S2 Table — Total number of positions acquired for each of four individual Short-toed Treecreepers in Copenhagen, Denmark, the calculated 100% minimum convex polygon (MCP) of known area use, and total area of ‘urban park’ and ‘road’ (calculated from Open StreetMap land use) within. Minimum Distance (Min. dist) between mature trees in park fragments used by each individual (as calculated from satellite images). (PDF) [file pone.0227731.s002.pdf]

S2 Table. Spatial data acquired for study birds

Year	Colour Ring	Dates	# pos	MCP (ha)	Urban Park (ha)	Road (ha)	Min. dist (m)
2016	Red	11-19 Feb	25	10.2	6.5	2.6	80
	Lime Green	22-29 Feb	5	9.8	7.5	2.0	200
	Yellow	22-26 Feb	18	20.6	7.7	7.4	91
2017	Pale Blue	6-8 Apr	9	10.0	5.8	2.5	32
	Lime Green	25 Jan-30 Apr	5	NA	NA	NA	80

Total number of positions acquired for each of four individual Short-toed Treecreepers in Copenhagen, Denmark, the calculated 100% minimum convex polygon (MCP) of known area use, and total area of ‘urban park’ and ‘road’ (calculated from Open StreetMap land use) within. Minimum Distance (Min. dist) between mature trees in park fragments used by each individual (as calculated from satellite images).
